# Supplementary figures and images for: Integrated analysis of the transcriptome and metabolome of purple and green leaves of Tetrastigma hemsleyanum reveals gene expression patterns involved in anthocyanin biosynthesis
Source: PLoS One. 2020 Mar 9;15(3):e0230154. doi: 10.1371/journal.pone.0230154 (PMC7062267; doi:10.1371/journal.pone.0230154)

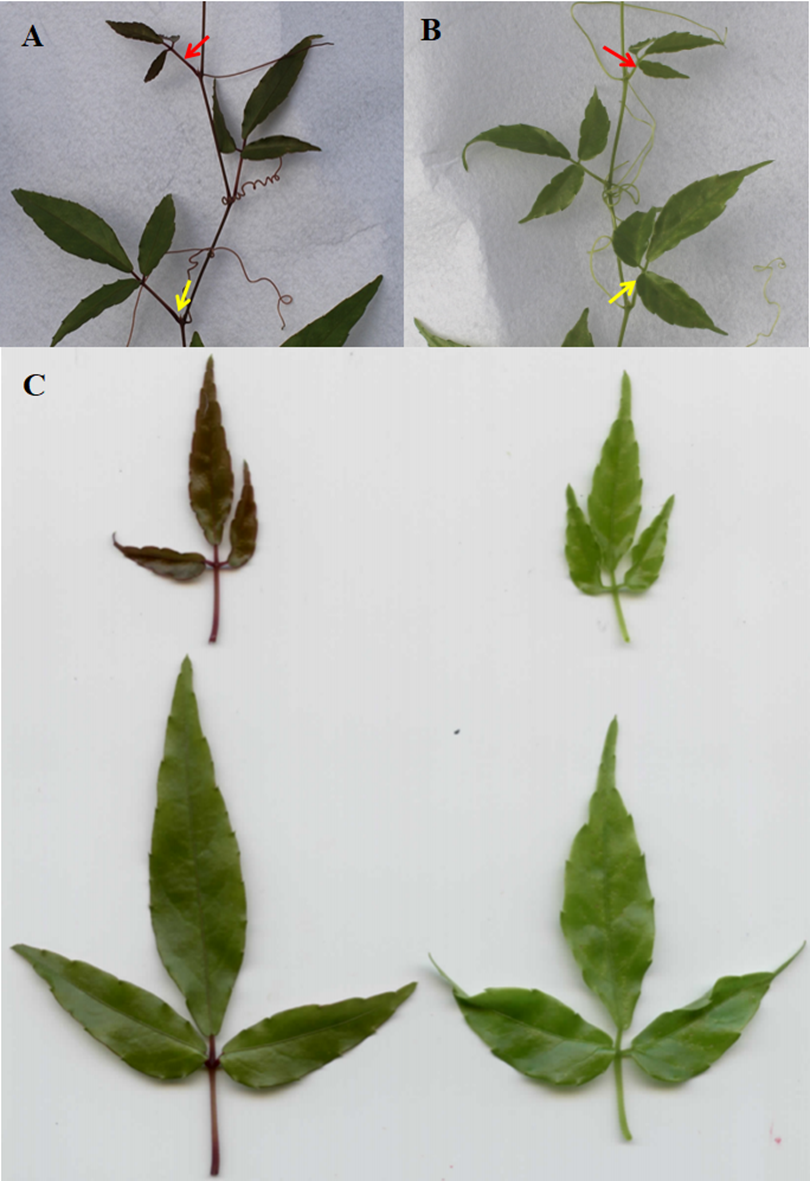

Supplement: S1 Fig — Red and yellow arrows indicate the leaves shown in S1 Fig. Yellow arrows indicate the position where the leaves were collected for further experiments. (TIFF) [file pone.0230154.s001.tiff]

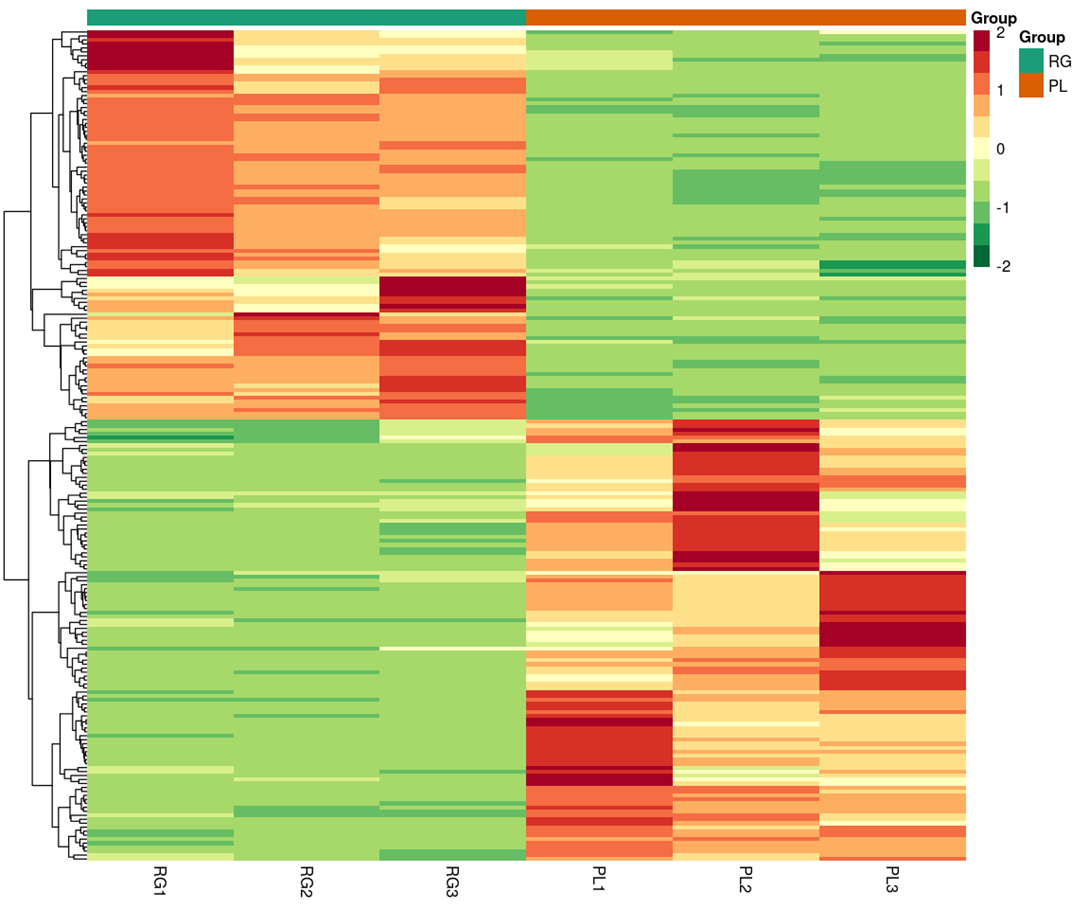

Supplement: S2 Fig — Normalized contents of different compounds are represented as colours ranging from green (-2) to red (2). (TIFF) [file pone.0230154.s002.tiff]
