# Supplementary material for: Integrated analysis of the transcriptome and metabolome of purple and green leaves of Tetrastigma hemsleyanum reveals gene expression patterns involved in anthocyanin biosynthesis
Source: PLoS One. 2020 Mar 9;15(3):e0230154. doi: 10.1371/journal.pone.0230154 (PMC7062267; doi:10.1371/journal.pone.0230154)
Supplement: Attachment — Submitted filename: MS revised.docx [file pone.0230154.s005.docx]

Integrated analysis of transcriptome and metabolome reveals gene expression pattern involved in anthocyanin biosynthesis in purple and green leaves of *Tetrastigma hemsleyanum*

**Jianli Yan^1^, Lihua Qian^1^, Weidong Zhu^2^, Jieren Qiu^1^, Qiujun Lu^4^, Xianbo Wang^1^, Qifeng Wu^5^, Songlin Ruan^1^*, Yuqing Huang^1^,^3^***

^1^ Laboratory of Plant Molecular Biology & Proteomics, Institute of Biotechnology, Hangzhou Academy of Agricultural Sciences, Hangzhou 310024, China

^2^ Quzhou Academy of Agricultural Sciences, Quzhou 324000, China

^3^ College of Agriculture and Biotechnology, Zhejiang University, Hangzhou 310058, China

^4^ Agricultural Science and Technology Education Terminal, Hangzhou Agricultural and Rural Bureau, Hangzhou 310026, China

^5^ Agriculture and Forestry Technology Promotion Center, Hangzhou Linan Agricultural and Rural Bureau, Hangzhou 311300, China

*****	Correspondence：[ruansl1@hotmail.com](mailto:ruansl1@hotmail.com); Tel.: +86-571-8709-3826 (S.R.); huangyq@zju.edu.cn; Tel.: +86-571-8709-3826 (Y.H.)

**Abstract**

To gain better insights into the regulatory networks of anthocyanin biosynthesis, an integrated analysis of metabolome and transcriptome in purple and green leaves of *Tetrastigma hemsleyanum* was conducted. Transcript and metabolite profiles were archived by RNA-sequencing data analysis and LC-ESI-MS/MS system, respectively. There were 209 metabolites and 4211 transcripts which were differentially expressed between purple and green leaves. Correlation tests of anthocyanin contents and transcriptional changes showed 141 significant correlations (Pearson correlation coefficient >0.8) between 16 compounds and 14 transcripts involved in anthocyanin biosynthesis pathway. Some novel genes and metabolites were discovered as potential candidate targets for the improvement of anthocyanin content and superior cultivars.

Introduction

*Tetrastigma hemsleyanum Diels et Gilg* (*T. hemsleyanum*, belonging to the family Vitaceae), also known as “Sanyeqing” (SYQ), is distributed in tropical to subtropical areas in Asia, mainly in the south China, such as Zhejiang, Guizhou and Guangxi provinces. The entire herb and its root tubers have been used as a broad-spectrum antibiotic material for treatment of fever, sore throat in Chinese for a long time. Previous findings have demonstrated that the principal and functional components of SYQ, such as flavonoids, polysaccharide and polyphenol, have various effect on anti-cancer, liver protection, and anti-inflammatory [1,2].

Flavonoids such as anthocyanins and flavanols were the most dominant and important gradient in SYQ [3,4], they are beneficial for human health, and also an important group of pigment which colour the leaves and flower in many plants [5]. Anthocyanins also involved in biotic and abiotic stress defending responses [6,7]. It is vital to elucidate the flavonoid biosynthesis and regulation in SYQ. Although several genes in anthocyanin regulation pathway have been identified [8], the unique regulation mechanism in SYQ remained unclear.

The color of the plants leaves is mainly determined by the composition and amount of the anthocyanins [9], the anthocyanins are the products of the branched flavonoid biosynthesis pathway. The accumulation of anthocyanins is regulated by the expression of the genes, including chalcone synthase (CHS), chalcone isomerase (CHI), flavanone 3-hydroxylase (F3H), flavonoid 3′-hydroxylase (F3'H), flavonoid 3′,5′-hydroxylase (F3'5'H), dihydroflavonol 4-reductase (DFR), anthocyanidin synthase (ANS) and flavonoid 3-O-glucosyltransferase (UFGT) [10].

In the past decade, RNA-sequencing has rapidly become an efficient approach to analyze the function of genes in a high throughput way [11]. The transcripts in low abundance or some unrevealed ever can be identified [12]. Advanced in metabolomics with the help of liquid chromatography / mass spectrometry (LC/MS) has been a powerful tool to discover a large number of compounds compared with traditional chemical analysis [13, 14]. Recently, the integrated analysis of metabolic and gene expression has been widely used in the exploitation network and correlation between metabolites and genes [15-17].

In this study, we explored the regulatory networks of flavonoid and anthocyanin biosynthesis in green (RG) and purple leaf (PL) SYQ at the levels of transcriptome and metabolome. The accumulation of different types of anthocyanin and the expression of related genes were investigated. The connection network was constructed to highlight the regulatory genes associated with metabolites. Our finding will provide insights into the accumulation mechanism of SYQ leaf color pigment and the regulation of anthocyanin biosynthesis.

Materials and Methods

**Plant materials and growth conditions**

The plants of green leaves (RG) and purple leaves (PL) genotypes (S1 Fig) of *Tetrastigma hemsleyanus* (Sanyeqing) were grown in the plant garden of Hangzhou Academy of Agricultural Sciences (Hangzhou, Zhejiang Province, China). Leaves at the third node away from the top were collected, frozen in liquid nitrogen and storage at -80℃ for RNA and metabolites isolation.

**RNA isolation and transcriptome sequencing**

Total RNA was isolated with TRIzol reagent (Invitrogen, USA) according to the manufacturer's protocol. The transcriptome sequence library was constucted using NEBNext Ultra RNA Library Prep Kits for Illumina (NEB, USA). Sequencing was performed on an Illumina HiSeq 2500 platform (Novogene, China). The reads were aligned to the genome using the TopHat (2.0.9) software after removing the reads containing adapter or poly-N and the low-quality reads from raw data. Total number of reads per kilobase per million reads (RPKM) of each gene was calculated based on the length of this gene and the counts of reads mapped to this gene. GO annotation was implemented using the Blast2GO software. KOBAS (2.0) software was used for KEGG enrichment analysis of differentially expressed genes.

**Metabolic profiling**

The samples were freeze-dried and crushed into power for metabolites isolation. One hundred milligram of each sample was extracted 1.0 ml 70% aqueous methanol at 4℃ for overnight. The extracts were absorbed (CNWBOND Carbon-GCB SPE Cartridge, 250mg, 3ml; ANPEL, Shanghai, China, www.anpel.com.cn/cnw) and filtrated (SCAA-104, 0.22μm pore size; ANPEL, Shanghai, China, http://www.anpel.com.cn/) before LC-MS analysis.

The sample extracts were analyzed using an LC-ESI-MS/MS system (HPLC, Shim-pack UFLC SHIMADZU CBM30A system, www.shimadzu.com.cn/; MS, Applied Biosystems 6500 Q TRAP, www.appliedbiosystems.com.cn/). A Waters ACQUITY UPLC HSS T3 C18 (1.8 µm, 2.1 mm*100 mm) was used for compound separation. The analytical conditions were set as follows: solvent system, water (0.04% acetic acid): acetonitrile (0.04% acetic acid); gradient program, 95:5V/V at 0 min, 5:95V/V at 11.0 min, 5:95V/V at 12.0min, 95:5V/V at 12.1min, 95:5V/V at 15.0 min; flow rate, 0.40 ml/min; temperature, 40°C; injection volume: 2 μl. Mass data acquisition was performed in both positive and negative modes using the following parameters: ion source, turbo spray; source temperature 500°C; ion spray voltage (IS) 5500 V; ion source gas I (GSI), gas II(GSII), curtain gas (CUR) were set at 55, 60, and 25.0 psi, respectively; the collision gas(CAD) was high. The mass fragmentations were compared to databases of HMDB (http://www.hmdb.ca), METLIN (http://metlin.scrippps.edu) and KEGG (http://kegg.jp). The obtained data were devoted to SIMCA-P V12.0.0 Demo (Umetric, Umea, Sweden) for principal components analysis (PCA) and partial least-squares discriminant analysis (PLS-DA).

**Verification of candidate genes by quantitative real-time PCR (qRT-PCR)**

The expression levels of transcripts involved in anthocyanin biosynthesis pathways were validated by qPCR using the identical RNA samples used for sequencing. The primers were designed using beacon designer 7.8 software, cDNAs were synthesized using SuperScript™ III First-Strand Synthesis SuperMix for qRT-PCR (Invitrogen). Power SYBR® Green PCR Master Mix (Applied Biosystems) was selected for the identification of the PCR products on a CFX384 Real-time PCR system (Bio-Rad). Three replicates were done for each sample and the primers of each sample were listed in Table S1.

Results

**Metabolic differences in Green Leaves and Purple Leaves**

To compare the metabolite composition in the two accessions, LC-MS was used for metabolite identification. Totally, 597 metabolites with known structures were detected in RG and PL. Differentially expressed proteins were defined as those with a variable importance for projection (VIP) value >1.5 compared to RG (Fig 1). Among the detected metabolites, 111 metabolites were up-regulated and 98 metabolites were down-regulated. The top 10 most differentially accumulated metabolites were listed in Table 1. The top 10 up-accumulated metabolites were mainly anthocyanins, flavone C-glycosides. Compared to RG, cyanidin 3-O-rutinoside showed the highest fold change in PL, with a log2 fold change (FC) (PL/RG) value of 20.59. For the 10 most down-accumulated metabolites, quinic acid O-glucuronic acid had the lowest log2 fold change (FC) (PL/RG) value of -21.73.

**Fig 1.VIP (variable importance for projection) plot of differentially accumulated metabolites in PL vs RG. The red dots were up-accumulated metabolites and green dots were down-accumulated metabolites.**

**Table 1. List of top 10 up- and down-accumulated metabolites in PL vs RG. Differentially expressed proteins were defined as those with a variable importance for projection (VIP) value >1.5.**

| **Index** | **Compounds** | **Class** | **RG1** | **RG2** | **RG3** | **PL1** | **PL2** | **PL3** | **VIP** | **Fold_Change** | **Log2FC** |
| --- | --- | --- | --- | --- | --- | --- | --- | --- | --- | --- | --- |
| pme1773 | Cyanidin 3-O-rutinoside (Keracyanin) | Anthocyanins | 9 | 9 | 9 | 19500000 | 11500000 | 11700000 | 1.363135 | 1581481.481 | 20.59285 |
| pmb0675 | C-pentosyl-apigenin O-p-coumaroylhexoside | Flavone C-glycosides | 9 | 9 | 9 | 4880000 | 18500000 | 819000 | 1.351613 | 896259.2593 | 19.77356 |
| pme3038 | 5-oxoproline | Amino acid derivatives | 9 | 9 | 9 | 2880000 | 7300000 | 2470000 | 1.362247 | 468518.5185 | 18.83775 |
| pme1793 | Pelargonin | Anthocyanins | 9 | 9 | 9 | 2760000 | 2500000 | 3770000 | 1.363342 | 334444.4444 | 18.35141 |
| pmb0837 | Procyanidin A3 | Proanthocyanidins | 9 | 9 | 9 | 3160000 | 1610000 | 2300000 | 1.362756 | 261851.8519 | 17.99839 |
| pmb3074 | 3-O-p-Coumaroyl quinic acid | Quinate and its derivatives | 9 | 9 | 9 | 1450000 | 1170000 | 1030000 | 1.363438 | 135185.1852 | 17.04458 |
| pme3300 | Tricetin | Flavone | 9 | 9 | 9 | 2510000 | 311000 | 819000 | 1.355341 | 134814.8148 | 17.04062 |
| pmb0808 | 6,7-dihydroxycoumarin 6-O-quinic acid | Coumarins | 9 | 9 | 9 | 1450000 | 9 | 1460000 | 0.958286 | 107778.1111 | 16.7177 |
| pme1816 | Neochlorogenic acid (5-O-Caffeoylquinic acid) | Quinate and its derivatives | 9 | 9 | 9 | 773000 | 868000 | 1050000 | 1.363533 | 99666.66667 | 16.60482 |
| pmb0749 | O-Feruloyl quinic acid | Quinate and its derivatives | 9 | 9 | 9 | 173000 | 546000 | 1510000 | 1.355143 | 82555.55556 | 16.33308 |
| pmb3058 | Quinic acid O-glucuronic acid | Quinate and its derivatives | 33800000 | 30800000 | 29200000 | 9 | 9 | 9 | 1.363641 | 2.88E-07 | -21.7282 |
| pme0398 | Chlorogenic acid (3-O-Caffeoylquinic acid) | Quinate and its derivatives | 22800000 | 17200000 | 16900000 | 9 | 9 | 9 | 1.363516 | 4.75E-07 | -21.007 |
| pmb0639 | 8-C-hexosyl-apigenin O-hexosyl-O-hexoside | Flavone C-glycosides | 14500000 | 8060000 | 24600000 | 9 | 9 | 9 | 1.362386 | 5.73E-07 | -20.7362 |
| pma6647 | C-hexosyl-chrysoeriol O-hexoside | Flavone C-glycosides | 6340000 | 5840000 | 6310000 | 9 | 9 | 9 | 1.363675 | 1.46E-06 | -19.3854 |
| pmb0652 | C-hexosyl-apigenin O-pentoside | Flavone C-glycosides | 6300000 | 5470000 | 5240000 | 9 | 9 | 9 | 1.363606 | 1.59E-06 | -19.265 |
| pmb1108 | Luteolin 6-C-hexoside 8-C-hexosyl-O-hexoside | Flavone C-glycosides | 1530000 | 382000 | 11500000 | 9 | 9 | 9 | 1.346659 | 2.01E-06 | -18.9221 |
| pmb0623 | 6-C-hexosyl chrysoeriol O-hexoside | Flavone C-glycosides | 2360000 | 1200000 | 5440000 | 9 | 9 | 9 | 1.360537 | 0.000003 | -18.3466 |
| pma6516 | C-hexosyl-apigenin O-hexosyl-O-hexoside | Flavone C-glycosides | 2710000 | 1850000 | 2170000 | 9 | 9 | 9 | 1.363427 | 4.01E-06 | -17.9273 |
| pmb0629 | Chrysoeriol 6-C-hexoside | Flavone C-glycosides | 2590000 | 1940000 | 1210000 | 9 | 9 | 9 | 1.362641 | 4.70E-06 | -17.6977 |
| pmb4777 | 4-Hydroxy-7-methoxycoumarin-beta-rhamnoside | Coumarins | 584000 | 856000 | 851000 | 9 | 9 | 9 | 1.363425 | 1.18E-05 | -16.3727 |

All differentially accumulated metabolites were subjected to KEGG analysis (Fig 2), metabolites participated in flavonoid biosynthesis, flavone and flavanol biosynthesis, tryptophan metabolism and biosynthesis of alkaloids derived from shikimate pathway were predominantly enriched.

Principal component analysis (PCA) was employed to reveal the differences in metabolites profiles among samples (Fig 3). The results showed that the first principal component (PC1, 52.98% of the total variables) were clearly separated in RG and PL samples, indicating that the accumulation patterns of metabolites are different in green and purple leaves.

**Fig 2. KEGG Pathway enrichment analysis based on the all differentially accumulated metabolites of SYQ purple and green leaves.**

**Fig 3. PCA score plot of purple and green leaves of SYQ.**

**Transcriptome analysis in Green Leaves and Purple Leaves**

After removing the low-quality reads, around 30,000,000 reads of each sample were generated. De novo transcriptome assembly using Trinity built 205,387 transcripts and 100,540 unigenes for the samples, the average length of the transcripts 1151bp. The unigenes were mapped to NR database, and then applied to Pfam database for annotation.

Using a false discovery rate (FDR) <0.01, log2FC>1 as threshold values, 4211 genes were found to be differentially expressed in PL vs. RG. Among them, 2035 genes were up-regulated and 2176 genes were down-regulated (Fig 4).

**Fig 4. Volcano plot of differentially regulated genes in PL vs RG. The red dots were up-regulated genes and green dots were down- regulated genes.**

Differentially expressed genes potentially involved in anthocyanin biosynthesis were identified. These included *CHS*, *CHI*, *F3H*, *F3'H*, *F3'5'H*, *DFR*, *ANS* and *UFGT*.

The enriched GO (Gene Ontology) terms for DEGs were analyzed (Fig 5). In biology process (BP) categories, the most significant terms were metabolic process, cellular process, single-organism process. In molecular function (MF) and cellular component (CC) categories, binding and catalytic activity, cell and cell part were mostly enriched respectively.

**Fig 5. GO classification of all DEGs of purple and green SYQ leaves. The above is up-regulated genes (A) and the below is down-regulated genes (B).**

The identified genes were mapped to KEGG reference pathways, they could be mapped onto 105 KEGG pathways (Fig 6). These pathways included flavonoid biosynthesis (ko00941), purine metabolism (ko00230) and so on, and plant pathogen interaction (ko04626) and carbon metabolism (ko01200) had the highest enrich factor values. In the flavonoid biosynthesis pathway, most genes were up-regulated in the purple leaves.

**Fig 6. KEGG pathway enrichment analysis based on the all DEGs of SYQ purple and green leaves. The above is up-regulated genes (A) and the below is down-regulated genes (B).**

Transcription factors are essential regulators in anthocyanin biosynthesis. A total of 176 TFs were identified, which were either up- or down-regulated between purples leaves and green leaves. As the most extensively studied TFs, one MYB and six bHLH were significantly different between purple and green leaves. MYB was highly expressed in purple leaves and four bHLH were up-regulated and two were down-regulated.

**Network analysis of metabolites and transcripts in SYQ**

The identified anthocyanins, their relevant compounds and the related genes were mapped onto their corresponding position in anthocyanin pathway. It indicated that the compounds in this pathway were different in purple and green leaves. The abundances of the transcripts and composition of the compounds were shown by the heatmap (Fig 7).

**Fig 7. Biosynthetic pathway of anthocyanins. This pathway is constructed based on the KEGG pathway. Each colored cell represents the normalized intensity of each compound ion according to the color scale (three biological replicates×two cultivars, n=6). The expression levels of chalcone synthase (CHS), chalcone isomerase (CHI), flavanone 3-hydroxylase (F3H), flavonoid 3′-hydroxylase (F3'H), flavonoid 3′,5′-hydroxylase (F3'5'H), dihydroflavonol 4-reductase (DFR), anthocyanidin synthase (ANS) and flavonoid 3-O-glucosyltransferase (UFGT) were shown.**

As was shown in Fig 7, pelargonidin, dihydrokaempferol, pelargonidin 3-O-glucoside were significantly accumulated in purple leaves, and dihydromyricetin, cyanidin, cyanidin 3-glucoside had higher levels in green leaves. A majority of the transcripts were promoted in purple leaves, for instance, the expressions of *CHS* and *CHI* were inhibited while the others were upregulated. In our results, the expression levels of the transcripts and the metabolites in pelargonidin biosynthesis pathway were increased.

To gain a better understanding of the regulatory network of the leaf color in the two accessions, the Pearson correlation test between the metabolites and the transcripts were carried out. In total, 14 genes and 16 metabolites which were involved in flavonoid and anthocyanin pathways were subjected to Pearson correlation analysis (Fig 8). There were 141 significant correlation combinations between the genes and metabolites which had a Pearson correlation coefficient >0.8 and p value<0.05.

**Fig 8. Connection network between regulatory genes and anthocyanin-related metabolites. The networks between metabolites and transcripts were visualized with the Cytoscape software (version 2.8.2).**

The expression levels of the key genes in anthocyanin biosynthesis pathway were analyzed by real-time quantitative PCR (qPCR). As was shown in Fig 9, *UGFT*, *ANS*, *DFR*, *F3’5’H*, *F3H* accumulated at a much higher level in the purple leaves, whereas the expression of *CHI* was reduced in purple leaves. The expression pattern of the genes involved in anthocyanin biosynthesis pathway revealed by qPCR was consistent with the RNA-seq results.

**Fig 9. PCR analysis of anthocyanin biosynthesis related gene expression green leaves and purple leaves SYQ cultivars.**

Discussion

In this study, the differences of transcripts and metabolites in green and purple leaves were compared. Using the ultra-performance liquid chromatography and tandem mass spectrometry, 597 metabolites were detected (Fig 1). As was shown in hierarchical clustering of the samples, there was a clear separation between purple leaves and green leaves (S2 Fig). In the differential accumulated metabolites, six metabolites annotated to anthocyanins were up-accumulated in purple leaves, only two anthocyanin compounds were inhibited.

To investigate the genes regulating the differential pigmentation in the leaves of SYQ, transcriptome profiling was engaged. Anthocyanin accumulation corresponds to the expression of the genes involved in the biosynthesis pathway [5,18]. Our study here showed that most of the key genes in anthocyanin biosynthesis pathway were up-regulated in purple leaves (Fig 7), indicating that the anthocyanin pathway was promoted in purple leaves. This founding was consistent with the gene expression results of the key genes in anthocyanin biosynthesis pathway (Fig 9). The largest log2FC (purple/green) value was found in *F3H*, which had a value of 5.869. F3H catalyzes the conversion of flavanone into dihydroflavanol [19]. The up-regulation of *F3H* facilitates the transition to anthocyanins.

There are many types of anthocyanins, cyanidin, delphinidin and pelargonidin have been recognized as the most common anthocyanins in plants. Different distribution of anthocyanins confers to different colors in fruits and leaves. For instance, the major type of anthocyanins in berries was cyanidin [20], red pigment was generated from the accumulation of pelargonidin [21] and delphinidin gives purple to plants. In our results, the biosynthesis pathway of pelargonidin-3-glucoside was promoted at both transcriptional and metabolic levels. The genes and metabolites worked coordinately to regulate the production of anthocyanins.

Transcription factors including MYB, bHLH and WD40 played vital roles in the regulation of flavonoid and anthocyanin pathway [22-24]. Only one of MYB members had higher expression level in purple leaves and different bHLHs transcripts were differentially expressed in different leaves. There are 8 WRKYs which showed higher expression levels in purple leaves. As suggested earlier, WRKY may be involved in the regulation of anthocyanin biosynthesis. More anthocyanin was accumulated in the *WRKY* overexpressed lines compared to wild type in *Arabidopsis* [25].

Polysaccharides, phenolic acids and flavanols are the main compound in SYQ with various biological activities which are beneficial for the cure of some disease. Although the bioactive compounds in root tubers of SYQ is abundant [26], the aerial parts of SYQ may be more practical due to its easily access. In our results, the composition of the bioactive compounds in purple leaves was higher in green leaves, in which may contain higher levels of antioxidant activity compared to the green leaves. Therefore, purple leaves with high anthocyanin content has potential as a superior cultivar with enhanced health benefits.

The network analysis of the metabolites and the transcripts were conducted. There were 141 significant correlation combinations between the 14 genes and 16 metabolites. Transcript c90950.graph_c0 (CHS) was dramatically correlated with 14 metabolites, which was the most (Fig 8). CHS is the initial enzyme of flavonoid biosynthesis, it catalyzes the synthesis of naringenin chalcone from 4-coumaroyl-CoA and malonyl-CoA. In different plant species, the expression pattern of CHS of red tissues and green tissues were different [27]. The expression level of *CHS* was found to be significantly upregulated in the red tomato fruits, while in the tomato mutants, the *CHS* expression was similar between the pigmented and non-pigmented ones [28]. Data obtained indicated that the metabolites and genes work coordinately to regulate the biosynthesis of anthocyanins. The regulatory genes which were highly correlated with the accumulation of metabolites were identified, they could provide new insights into the regulatory mechanism of the anthocyanin biosynthesis.

Conclusions

We analyzed the regulatory network of anthocyanin biosynthesis with integration of metabolome and transcriptome in purple and green leaves of SYQ. Correlation analysis of the metabolites and transcripts involved in anthocyanin biosynthesis pathway was conducted. A regulatory network of metabolites and transcripts related to leaf color could be resources for the exploitation of the mechanism of anthocyanin regulation in SYQ.

**Author Contributions**

J.Y. carried out analysis of transcriptomics, bioinformatics and Q-PCR. L.Q., W.Z. and J.Q. participated in metabolomics and bioinformatics. X.W. and Q.L.carried out material preparation and phenotypic analysis. S.R. and Y.H. conceived of the study, participated in its design and coordination and completed the manuscript. All authors read and approved the final manuscript.

**Funding**

This work was sponsored by grants Active Design Project of Hangzhou Agricultural and Social Development Scientific Research (No. 20190101A05), and Science and Technology Innovation and Demonstration extension Fund of Hangzhou Academy of Agricultural Sciences (No. 2019HNCT-31).

**Acknowledgments**

This research was supported by the Grants from the Active Design Project of Hangzhou Agricultural and Social Development Scientific Research (No. 20190101A05), and Science and Technology Innovation and Demonstration extension Fund of Hangzhou Academy of Agricultural Sciences (No. 2019HNCT-31).

**Conflicts of Interest**

The authors declare that they have no competing interests.

References

1.	Huang Z, Mao Q-q, Wei J-p. Evaluation of anti-inflammatory, analgesic and antipyretic actions for the extracts from Radix Tetrastigmae. Chinese New Drugs Journal. 2005;14(7):861.

2.	Dong L, jianhua J, geng L, xudong X, junshan Y, guagnzhong T. New C-glycosylflavones from Tetrastigma hemsleyanum (Vitaceae). Acta Botanica Sinica. 2002;44(2):227-9.

3.	Sun Y, Liu J, Kennedy JF. Application of response surface methodology for optimization of polysaccharides production parameters from the roots of Codonopsis pilosula by a central composite design. Carbohydrate Polymers. 2010;80(3):949-53.

4.	Yongjiang W, Zhong C, Jianwei M, Minger F, Xueqian W. Optimization of ultrasonic-assisted extraction process of Poria cocos polysaccharides by response surface methodology. Carbohydrate Polymers. 2009;77(4):713-7.

5.	Tanaka Y, Ohmiya A. Seeing is believing: engineering anthocyanin and carotenoid biosynthetic pathways. Current opinion in biotechnology. 2008;19(2):190-7.

6.	Chalker‐Scott L. Environmental significance of anthocyanins in plant stress responses. Photochemistry and photobiology. 1999;70(1):1-9.

7.	Ahmed NU, Park J-I, Jung H-J, Yang T-J, Hur Y, Nou I-S. Characterization of dihydroflavonol 4-reductase (DFR) genes and their association with cold and freezing stress in Brassica rapa. Gene. 2014;550(1):46-55.

8.	Passeri V, Koes R, Quattrocchio FM. New challenges for the design of high value plant products: stabilization of anthocyanins in plant vacuoles. Frontiers in plant science. 2016;7:153.

9.	Baranac JM, Petranović NA, Dimitrić-Marković JM. Spectrophotometric study of anthocyan copigmentation reactions. 3. Malvin and the nonglycosidized flavone morin. Journal of agricultural and food chemistry. 1997;45(5):1698-700.

10.	Salvatierra A, Pimentel P, Moya-Leon MA, Caligari PD, Herrera R. Comparison of transcriptional profiles of flavonoid genes and anthocyanin contents during fruit development of two botanical forms of Fragaria chiloensis ssp. chiloensis. Phytochemistry. 2010;71(16):1839-47.

11.	Shendure J, Ji H. Next-generation DNA sequencing. Nature biotechnology. 2008;26(10):1135.

12.	Wang Z, Gerstein M, Snyder M. RNA-Seq: a revolutionary tool for transcriptomics. Nature reviews genetics. 2009;10(1):57.

13.	Fernie AR, Trethewey RN, Krotzky AJ, Willmitzer L. Metabolite profiling: from diagnostics to systems biology. Nature reviews molecular cell biology. 2004;5(9):763.

14.	Büscher JrM, Czernik D, Ewald JC, Sauer U, Zamboni N. Cross-platform comparison of methods for quantitative metabolomics of primary metabolism. Analytical chemistry. 2009;81(6):2135-43.

15.	Urbanczyk‐Wochniak E, Luedemann A, Kopka J, Selbig J, Roessner‐Tunali U, Willmitzer L, et al. Parallel analysis of transcript and metabolic profiles: a new approach in systems biology. EMBO reports. 2003;4(10):989-93.

16.	Hoefgen R, Nikiforova VJ. Metabolomics integrated with transcriptomics: assessing systems response to sulfur‐deficiency stress. Physiologia Plantarum. 2008;132(2):190-8.

17.	Saito K, Hirai MY, Yonekura-Sakakibara K. Decoding genes with coexpression networks and metabolomics–‘majority report by precogs’. Trends in plant science. 2008;13(1):36-43.

18.	Holton TA, Cornish EC. Genetics and biochemistry of anthocyanin biosynthesis. The Plant Cell. 1995;7(7):1071.

19.	Bogs J, Ebadi A, McDavid D, Robinson SP. Identification of the flavonoid hydroxylases from grapevine and their regulation during fruit development. Plant Physiology. 2006;140(1):279-91.

20.	Seeram NP, Momin RA, Nair MG, Bourquin LD. Cyclooxygenase inhibitory and antioxidant cyanidin glycosides in cherries and berries. Phytomedicine. 2001;8(5):362-9.

21.	B¹kowska-Barczak A. Acylated anthocyanins as stable, natural food colorants–a review. Polish journal of food and nutrition sciences. 2005;14:55.

22.	Koes R, Verweij W, Quattrocchio F. Flavonoids: a colorful model for the regulation and evolution of biochemical pathways. Trends in plant science. 2005;10(5):236-42.

23.	Winkel-Shirley B. Flavonoid biosynthesis. A colorful model for genetics, biochemistry, cell biology, and biotechnology. Plant physiology. 2001;126(2):485-93.

24.	Springob K, Nakajima J-i, Yamazaki M, Saito K. Recent advances in the biosynthesis and accumulation of anthocyanins. Natural product reports. 2003;20(3):288-303.

25.	Lin-Wang K, McGhie TK, Wang M, Liu Y, Warren B, Storey R, et al. Engineering the anthocyanin regulatory complex of strawberry (Fragaria vesca). Frontiers in plant science. 2014;5:651.

26.	Li Y, Lu W, Yu Z. Studies of chemical component in Tetrastigma hemsleyanum. Chin Tradit Herb Drugs. 2003;34:982-3.

27.	Povero G, Gonzali S, Bassolino L, Mazzucato A, Perata P. Transcriptional analysis in high-anthocyanin tomatoes reveals synergistic effect of Aft and atv genes. Journal of plant physiology. 2011;168(3):270-9.

28.	Borovsky Y, Oren-Shamir M, Ovadia R, De Jong W, Paran I. The A locus that controls anthocyanin accumulation in pepper encodes a MYB transcription factor homologous to Anthocyanin2 of Petunia. Theoretical and Applied Genetics. 2004;109(1):23-9.

**Supporting information**

S1 Fig. Phenotypes of purple-leaf SYQ (A-PL) and green-leaf SYQ (B-RG). Red and yellow arrows indicated the leaves shown in S1 Fig. Yellow arrows indicate the consistent position where the leaves were collected for further experiments.

S2 Fig. Hierarchical clustering of differentially accumulated metabolites PL and RG samples. Normalized contents of different compounds are represented as colors ranging from green (-2) to red (2).

Table S1. Sequence information for primers.
