# Supplementary material for: Integrated analysis of the transcriptome and metabolome of purple and green leaves of Tetrastigma hemsleyanum reveals gene expression patterns involved in anthocyanin biosynthesis
Source: PLoS One. 2020 Mar 9;15(3):e0230154. doi: 10.1371/journal.pone.0230154 (PMC7062267; doi:10.1371/journal.pone.0230154)
Supplement: Attachment — Submitted filename: reviews-Zhigang Han.docx [file pone.0230154.s006.docx]

This manuscript applied metabolome and transcriptome to reveal the alteration of regulatory network in anthocyanin biosynthesis in SYQ. It is meaningful to discover some novel genes and metabolites for the improvement of anthocyanin content and superior cultivars. However, there are some problems on writing of this manuscript.

-Minor issues

Line 35-36, “Previous findings have demonstrated that the principal and functional components of SYQ, such as flavonoids, polysaccharide and polyphenol, have various effect on anti-cancer, liver protection, and anti-inflammatory”, I think polyphenol include flavonoids.

Line 40, “Anthocyanins also involved in biotic and abiotic stress defending responses”, rephrase this sentence.

Line 43-44, “The color of the plants leaves is mainly determined by the composition and amount of the anthocyanins, the anthocyanins are the products of the branched flavonoid biosynthesis pathway”, “amount” is not proper in this sentence.

Line 50, “The transcripts in low abundance or some unrevealed ever can be identified”, rephrase this sentence.

Line 51-52, “Advanced in metabolomics with the help of liquid chromatography / mass spectrometry (LC/MS) has been a powerful tool to discover a large number of compounds compared with traditional chemical analysis”, “Advanced in metabolomics”? Rephrase this sentence.

Line 203, check and rephrase this sentence.

Line 220-221, “Although the bioactive compounds in root tubers of SYQ is abundant, the aerial parts of SYQ may be more practical due to its easily access”, what are the aerial parts? And why are these parts easily access?

Line 223-224, check and rephrase this sentence.
