# Supplementary material for: Integrated analysis of the transcriptome and metabolome of purple and green leaves of Tetrastigma hemsleyanum reveals gene expression patterns involved in anthocyanin biosynthesis
Source: PLoS One. 2020 Mar 9;15(3):e0230154. doi: 10.1371/journal.pone.0230154 (PMC7062267; doi:10.1371/journal.pone.0230154)
Supplement: Attachment — Submitted filename: Responses to reviewers.docx [file pone.0230154.s007.docx]

**Responses to reviewers’ comments and suggestion**

First of all, we are deeply indebted to your careful examination and valuable comments for further improvement of this manuscript. Accordingly, we revised the MS according to your comments and suggestion. The detail revisions are as follows.

From Vicky Stabler:

1.Please ensure that your manuscript meets PLOS ONE's style requirements, including those for file naming.

Au: Thank you for your comment. We have re-checked the style and file naming of our manuscript.

2.We suggest you thoroughly copyedit your manuscript for language usage, spelling, and grammar. If you do not know anyone who can help you do this, you may wish to consider employing a professional scientific editing service.

Au: We employed American Journal Experts (AJE) to polish our manuscript. The new version was submitted.

3.In the Introduction to your manuscript, please remove any claims regarding the medicinal benefits of *Tetrastigma hemsleyanum* extracts which are not substantiated by robust scientific and clinical evidence.

Au: Relevant information was deleted in the revised version.

4.In your Data Availability statement, you have not specified where the minimal data set underlying the results described in your manuscript can be found.

Au: A new supplementary table (Table S2) of the sequences of all DEGs was added.

5.PLOS requires an ORCID iD for the corresponding author in Editorial Manager on papers submitted after December 6th, 2016.

Au: The ORCID iD was validated in the submission system.

Reviewer #1: Please modify the manuscript according to the revised MS.

1.The author should describe the background of the two accessions of SYQ, and their relation.

Au: Relevant information was added in the revised version.

2. I comment on related issues and hope the authors find these helpful to polish their work before final publication.

Au: Thank you for your careful examination and valuable comments. In the revised version, the spelling and grammatical errors were corrected.

Reviewer #2: This manuscript applied metabolome and transcriptome to reveal the alteration of regulatory network in anthocyanin biosynthesis in SYQ. It is meaningful to discover some novel genes and metabolites for the improvement of anthocyanin content and superior cultivars. However, there are some problems on writing of this manuscript.

-Minor issues

1. Line 35-36, “Previous findings have demonstrated that the principal and functional components of SYQ, such as flavonoids, polysaccharide and polyphenol, have various effect on anti-cancer, liver protection, and anti-inflammatory”, I think polyphenol include flavonoids.

Au: Thank you for your comment. It was corrected in revised version.

Line 40, “Anthocyanins also involved in biotic and abiotic stress defending responses”, rephrase this sentence.

Au: We rewrote it in the revised version.

Line 43-44, “The color of the plants leaves is mainly determined by the composition and amount of the anthocyanins, the anthocyanins are the products of the branched flavonoid biosynthesis pathway”, “amount” is not proper in this sentence.

Au: Sorry we made an error. “amount” was changed to “concentration” in the revised version.

Line 50, “The transcripts in low abundance or some unrevealed ever can be identified”, rephrase this sentence.

Au: This sentence was rewritten in revised version.

Line 51-52, “Advanced in metabolomics with the help of liquid chromatography / mass spectrometry (LC/MS) has been a powerful tool to discover a large number of compounds compared with traditional chemical analysis”, “Advanced in metabolomics”? Rephrase this sentence.

Au: Thank you for your comment. “Advanced” was changed to “Advances” in the revised version.

Line 203, check and rephrase this sentence.

Au: Relevant correction was made in revised version.

Line 220-221, “Although the bioactive compounds in root tubers of SYQ is abundant, the aerial parts of SYQ may be more practical due to its easily access”, what are the aerial parts? And why are these parts easily access?

Au: “aerial parts” was changed to “shoots” in the revised version.

Line 223-224, check and rephrase this sentence.

Au: The correction was made in the revised version.
